# Overexpression of c‐Fos reverses osteoprotegerin‐mediated suppression of osteoclastogenesis by increasing the Beclin1‐induced autophagy

**DOI:** 10.1111/jcmm.16152

**Published:** 2020-12-04

**Authors:** Xishuai Tong, Miaomiao Chen, Ruilong Song, Hongyan Zhao, Jianchun Bian, Jianhong Gu, Zongping Liu

**Affiliations:** ^1^ Institutes of Agricultural Science and Technology Development Joint International Research Laboratory of Agriculture and Agri‐Product Safety of the Ministry of Education of China Yangzhou University Yangzhou China; ^2^ College of Veterinary Medicine Yangzhou University Yangzhou China; ^3^ Jiangsu Co‐innovation Center for Prevention and Control of Important Animal Infectious Diseases and Zoonoses Yangzhou China; ^4^ Jiangsu Key Laboratory of Zoonosis Yangzhou China; ^5^ Center of Excellence for Vector‐Borne Diseases Department of Diagnostic Medicine/Pathobiology College of Veterinary Medicine Kansas State University Manhattan KS USA

**Keywords:** autophagy, Beclin1, c‐Fos, OPG, osteoclastogenesis

## Abstract

Osteoclastogenesis requires the involvement of transcription factors and degrading enzymes, and is regulated by upstream and downstream signalling. However, c‐Fos how regulates osteoclastogenesis through autophagy remain unclear. This study aimed to explore the role of c‐Fos during osteoprotegerin (OPG)‐mediated suppression of osteoclastogenesis. We found that the number of osteoclasts and the expression of c‐Fos, MMP‐9, CAⅡ, Src and p62 were decreased after treated with OPG, including attenuation the PI3K/Akt and the TAK1/S6 signalling pathways, but the expression of Beclin1 and LC3Ⅱ were increased. Knockdown of Beclin1 could reverse the expression of c‐Fos and MMP‐9 by activating the PI3K/Akt signalling pathway, but inhibiting the autophagy and the TAK1/S6 signalling pathway. In addition, inhibition of autophagy using the PI3K inhibitor LY294002 did not rescues OPG‐mediated suppression of osteoclastogenesis, but caused reduction of the expression of c‐Fos and CAⅡ by attenuating the autophagy, as well as the PI3K/Akt and the TAK1/S6 signalling pathways. Furthermore, continuous activation of c‐Fos could reverse OPG‐mediated suppression of osteoclastogenesis by activating the autophagy and the PI3K/Akt and the TAK1/S6 signalling pathways. Thus, overexpression of c‐Fos could reverse OPG‐mediated suppression of osteoclastogenesis via activation of Beclin1‐induced autophagy, indicating c‐Fos might serve as a new candidate for bone‐related basic studies.

## 1 INTRODUCTION

Osteoclast is tartrate‐resistant acid phosphatase (TRAP) abundant cell and is responsible for bone resorption to against bone formation. Osteoclastogenesis requires a series of transcription factors and degrading enzymes performing bone resorption.^1^ c‐Fos is a key regulatory molecule in receptor activator of nuclear factor‐κB ligand (RANKL)‐induced osteoclastogenesis.^2^ Knockout of *c‐Fos* gene in mice leads to severe osteopetrosis by inhibiting the osteoclastogenesis.^3^ Similarly, Src can phosphorylate the downstream signalling causing actin polymerization and ruffled border formation in osteoclastogenesis.^4^ In addition, a few certain degrading enzymes (eg matrix metalloproteinase‐9 [MMP‐9] and carbonic anhydrase II [CAⅡ]) are secreted by ruffled border in osteoclastogenesis. MMP‐9 is a key bone matrix‐related degrading enzyme and is involved in bone resorption within ruffled border.^5^, ^6^ Similarly, CAⅡ can sense the change of pH value by regulating the secretion of H^+^, which is responsible to osteoclastic bone resorption.^7^, ^8^

Autophagy can supply the energy for cells that is provided by self‐digestion and self‐degradation of damaged organelles, misfolded proteins or macromolecules.^9^ Similarly, osteoclast also undergo an autophagy process to maintain intracellular balance. In addition, adaptor protein p62 (SQSTM‐1) plays an essential role in RANKL‐induced osteoclastogenesis.^10^ Knockdown of p62 could attenuate autophagy causing accumulation of microtubule‐associated protein 1 light chain 3 (LC3) in RANKL‐induced osteoclastogenesis.^11^ LC3 is located at the membrane of autophagosomes and is also targeted to ruffled border of osteoclasts.^9^ Knockdown of LC3 did not affect the number of osteoclasts, but could suppress the release of cathepsin K (CSTK) enzyme and bone resorption activity by impairing the formation of actin in osteoclastogenesis.^12^

Besides, autophagy able to adapt to some exogenous or endogenous stresses to avoid cell death. Autophagy marker protein Beclin1 is interacts with the anti‐apoptotic multidomain proteins of the BCL‐2 family through binding to a BH3 domain (apoptosis‐inducing properties).^13^ Furthermore, inhibition of autophagy by knockdown of Beclin1 promoted cell apoptosis, while the suppression of caspases leads to activation of autophagy.^14^ Beclin1 is associated with the phosphoinositide 3‐kinase (PI3K)/vacuolar protein sorting 34 (Vps34) complex, which modulates autophagosomes formation in the early stage of autophagy.^15^ Inhibition of PI3K can suppress the phosphorylation of protein kinase B (PKB, also known as Akt) leading to the activation of nuclear factor‐κB (NF‐κB), thereby causing rapidly apoptosis of osteoclasts.^16^ In addition, TGF‐activated kinase 1 (TAK1) is a positive regulator and is related to osteoclastic function.^17^ Knockout of TAK1 mice exhibited normal body weight, limb size and fertility; however, it showed severe osteoporosis due to decrease the expression of c‐Fos and NF‐κB.^18^ Furthermore, TAK1‐deficient monocytes rescued from programmed cell death, but did not formation of osteoclasts in the present of RANKL.^19^

In this study, to examine the regulatory role of c‐Fos during OPG‐mediated suppression of osteoclastogenesis via Beclin1‐induced autophagy in vitro. Our data indicated that c‐Fos plays an indispensable role in OPG‐mediated suppression of osteoclastogenesis by regulating Beclin1‐induced autophagy, which depends upon the PI3K/Akt signalling pathway.

## 2 MATERIALS AND METHODS

### 2.1 Reagents

Foetal bovine serum (FBS), α‐minimum essential medium (α‐MEM) and Dulbecco's modified eagle's medium (DMEM) were obtained from Thermo Fisher Scientific (Waltham, MA, USA). The JetPRIME® transfection reagent was obtained from Polypus‐transfection (Strasbourg, France). The TRAP staining kit, chloroquine (CQ), antibodies against LC3B and p62, and LY294002 were obtained from Sigma Aldrich (St. Louis, MO, USA). RANKL, OPG and macrophage colony‐stimulating factor (M‐CSF) were obtained from R&D systems (Minneapolis, MN, USA). Antibodies against c‐Fos, Src, PI3K (p85), phosphorylated (p)‐Akt (Ser473), Akt, TAK1, Beclin1 and p‐S6 (Ser240/244) were obtained from Cell Signaling Technology (Danvers, MA, USA). Antibodies against MMP‐9, and CAⅡ were obtained from Abcam (Beverly, MA, USA). EGFP‐pmCherry‐LC3 plasmid was obtained from HedgehogBio Science and Technology Ltd. (Shanghai, China). Beclin1 siRNA plasmid (sc‐29798) was obtained from Santa Cruz Biotechnology (Dallas, TX, USA). BCA protein assay kit and rapamycin (Rap), was obtained from Beyotime (Beijing, China). All related reagents were available in our laboratory.

### 2.2 Cell culture

Five‐week‐old male BALB/c mice femurs and tibia were dissected, and bone marrow monocytes (BMMs) were separated from them by washing with α‐MEM. Animal experiment protocols were approved by the Animal Care and Use Committee of Yangzhou University (SYXK [Su] 2017‐0044). BMMs were cultured with α‐MEM (supplemented with 10% FBS) and M‐CSF (10 ng/mL) at 37°C, 5% CO_2_ for 12 hours. Non‐adherent cells were collected supplement with M‐CSF (30 ng/mL) and RANKL (60 ng/mL) to form osteoclasts (as primary osteoclasts). Mouse monocyte/macrophage cell line RAW264.7 cell was purchased from the Shanghai Institutes for Biological Sciences, Chinese Academy of Sciences (Shanghai, China). RAW264.7 cells are cultured with DMEM (supplemented with 10% FBS). DMEM medium was replaced by α‐MEM to induce the formation of osteoclast like cells (OCLs). All medium was replaced every 2 days.

### 2.3 Osteoclastogenesis

TRAP‐positive multinucleated cells were stained using the TRAP staining kit as previous described.^20^ Cells were fixed in 4% paraformaldehyde solution for 15 minutes at room temperature, and then incubated at 37°C according to kit manufacturer's instructions. Cells (≥ three nuclei) as differentiated or matured osteoclasts, which were captured randomly by the normal inverted microscope (Leica, Germany).

### 2.4 Cell transfection

EGFP‐pmCherry‐LC3 plasmid (500 ng/well) was transfected into cells using JetPRIME® transfection reagent for 24 hours. Cells were fixed in 4% paraformaldehyde solution for 15 minutes at room temperature. LC3 puncta were captured by the TCS SP8 STED high‐resolution laser confocal microscope (Leica, Germany).

Beclin1 siRNA plasmid sequences (sense 5′‐3′: GUACCGACUUGUUCCCUAUtt, antisense 5′‐3′: AUAGGGAACAAGUCGGUACtt), EGFP (pcDNA3.1‐EGFP) and c‐Fos (pcDNA3.1‐c‐fos) were transfected into cells using JetPRIME® transfection reagent for 24 hours. All medium was replaced by α‐MEM supplement with M‐CSF (30 ng/mL) and RANKL (60 ng/mL), and added the OPG (40 ng/mL) or not.

### 2.5 Immunoblotting

Total cellular protein was extracted, and protein concentration was quantified using a BCA protein assay kit. For immunoblotting, each sample (20‐30 μg/well) was separated by electrophoresis, transferred into PVDF membrane (Merck Millipore, Billerica, MA, USA). Membrane was blocked with 5% BSA for 1.5 hours at room temperature. Primary antibodies were incubated with different membranes at 4°C overnight. After washing, the secondary antibody was incubated with the membranes for 1.5 hours. Targeted proteins on the PVDF membranes were visualized by the 5200 Tanon ECL detection system (Shanghai, China). All bands were further quantitatively analysed.

### 2.6 Statistical analysis

All experimental data were analysed using one‐way analysis of variance (ANOVA) using SPSS 25.0 software (IBM Corp., Armonk, NY, USA). All experiments were performed more than three times.

## 3 RESULTS

### 3.1 OPG decreases osteoclastogenesis and the PI3K/Akt and TAK1/S6 signalling pathways by increasing the autophagy

**Figure 1:**
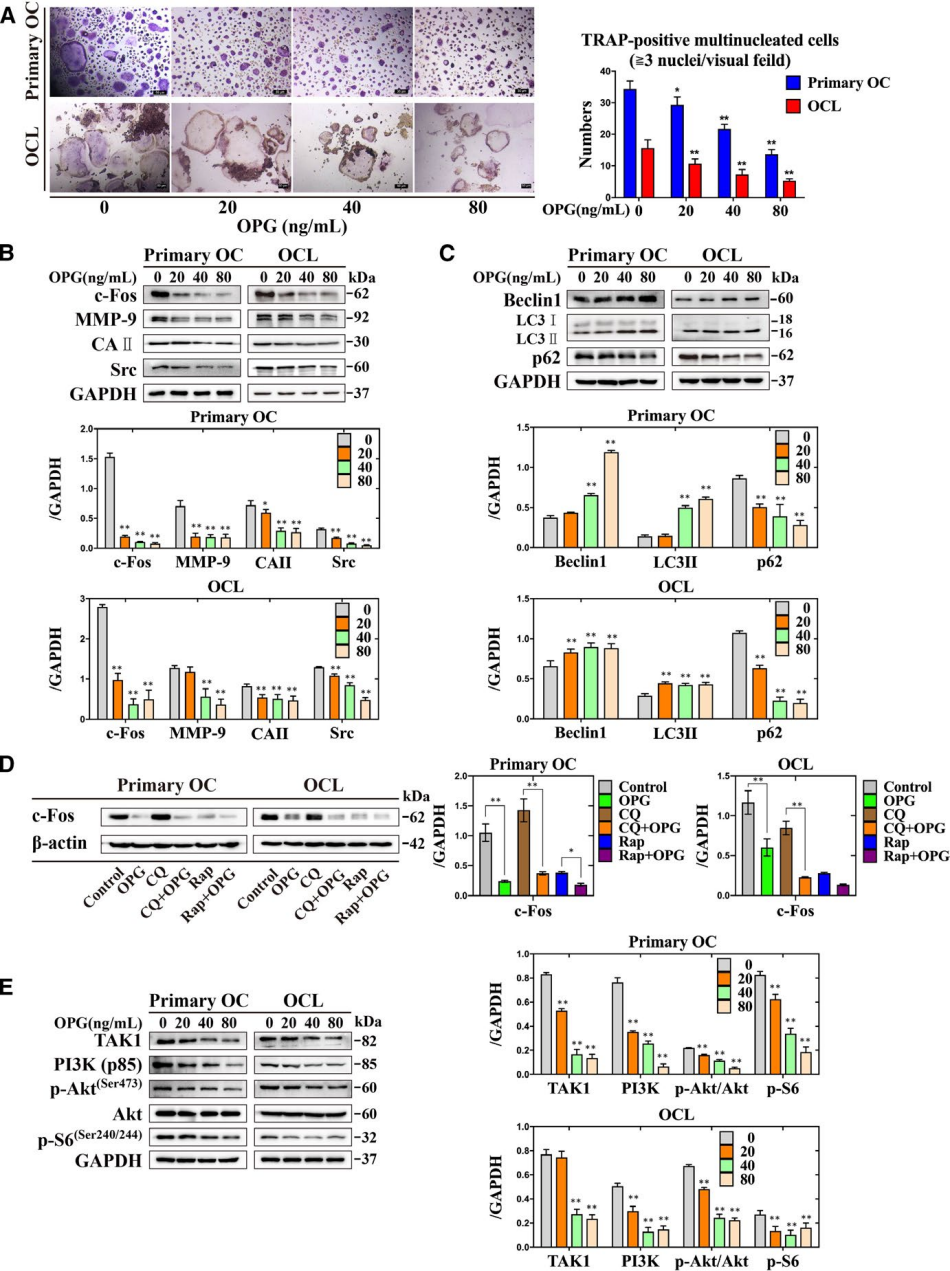
OPG inhibits osteoclastogenesis by increasing the autophagy, and inactivation of PI3K/Akt and TAK1/S6 signalling pathways in primary osteoclasts and OCLs. A, The formation and number of TRAP‐positive multinucleated cells were counted treatment with different concentrations of OPG. B‐E, The expression of c‐Fos, MMP‐9, CAⅡ, Src, Beclin1, LC3II, p62, the PI3K/Akt and the TAK1/S6 signalling‐related proteins were detected incubation with different treatments. All values are the mean ± SD. ^**^ *P* < .01, ^*^ *P* < .05

c‐Fos is a master transcription factor in osteoclastogenesis.^4^ Similarly, Src is a critical factor for the regulation of osteoclast cytoskeleton, while inactivation of Src leads to the down‐regulation of MMP‐9 and CAII mRNA.^21^ The number of TRAP‐positive multinucleated osteoclasts were decreased, the expression of c‐Fos, MMP‐9, CAII and Src were down‐regulated after treated with different concentration of OPG (Figure 1A and B). Autophagy is a programmed cell death process, including many signalling pathways that regulate osteoclastogenesis.^22^ Autophagy is also involved in OPG‐mediated suppression of osteoclastogenesis.^23^, ^24^ The expression of Beclin1 and LC3II were increased after treated with different concentration of OPG, but the expression of p62 was decreased (Figure 1C). Next, CQ‐only group has preserved the expression of c‐Fos, whereas CQ + OPG group decreased the expression of c‐Fos. However, there were decreased the expression of c‐Fos after treated with Rap‐only or Rap + OPG group (Figure 1D). Meanwhile, the expression of TAK1, PI3K (p85), p‐S6 (Ser240/244) and the ratio of p‐Akt (Ser473)/Akt were decreased after treated with different concentration of OPG (Figure 1E). These data indicated that OPG attenuates osteoclastogenesis by increasing the autophagy.

### 3.2 Knockdown of Beclin1 decreases autophagy by activating PI3K/Akt signalling pathway

**Figure 2:**
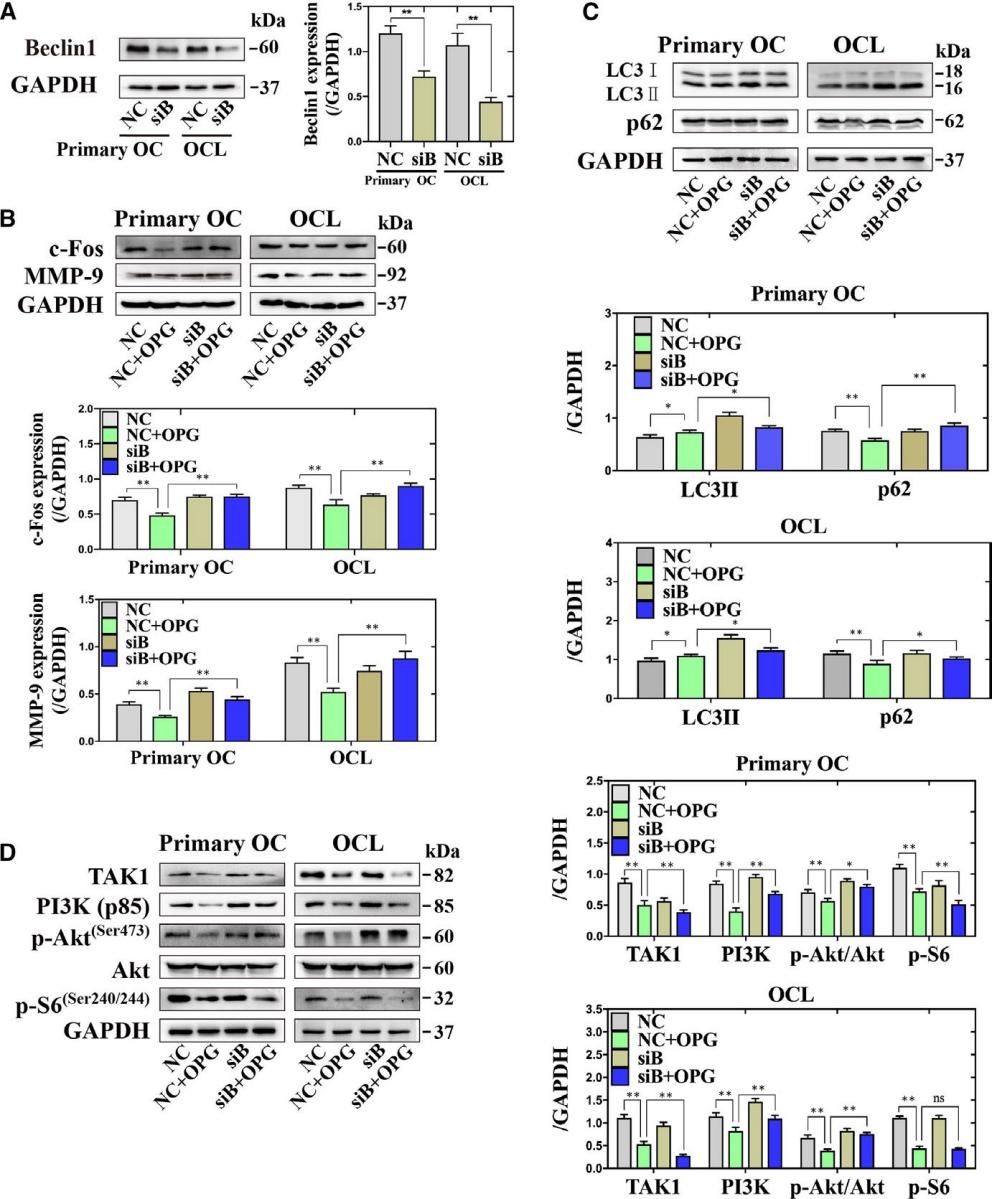
Knockdown of Beclin1 could reverse the expression of c‐Fos and MMP‐9 and the PI3K/Akt signalling pathway by reducing autophagy during OPG‐mediated suppression of osteoclastogenesis in primary osteoclasts and OCLs. A, The expression of Beclin1 was detected treatment with NC or knockdown of Beclin1. ^**^ *P* < .01 vs. NC. B‐D, The expression of c‐Fos and MMP‐9, LC3II, p62, the PI3K/Akt and the TAK1/S6 signalling‐related proteins were detected incubation with different treatments. All of values are mean ± SD. ^**^ *P* < .01, ^*^ *P* < .05

Beclin1 plays an important role in the early stage of autophagy, which is also involved in osteoclastogenesis.^25^ Besides, Beclin1 have a non‐autophagic role in RANKL‐induced osteoclastogenesis, and its activation by up‐regulating the expression of nuclear factor of activated T‐cells cytoplasmic 1 (NFATc1).^26^ The appropriate concentration of Beclin1 siRNA (siB) was selected the 20 nmol/L (Figure 2A). The expression of c‐Fos, MMP‐9, LC3II and p62 were increased by treated with siB + OPG group compared with NC + OPG group (Figure 2B and C). The expression of PI3K (p85) and the ratio of p‐Akt (Ser473)/Akt were increased, but the expression of TAK1 and p‐S6 were decreased by treated with siB + OPG group compared with NC + OPG group (Figure 2D). These data revealed that knockdown of Beclin1 could decrease autophagy through the PI3K/Akt signalling pathway during OPG‐mediated suppression of osteoclastogenesis.

### 3.3 Inhibition of PI3K can attenuate the autophagy in response to OPG‐mediated suppression of osteoclastogenesis

**Figure 3:**
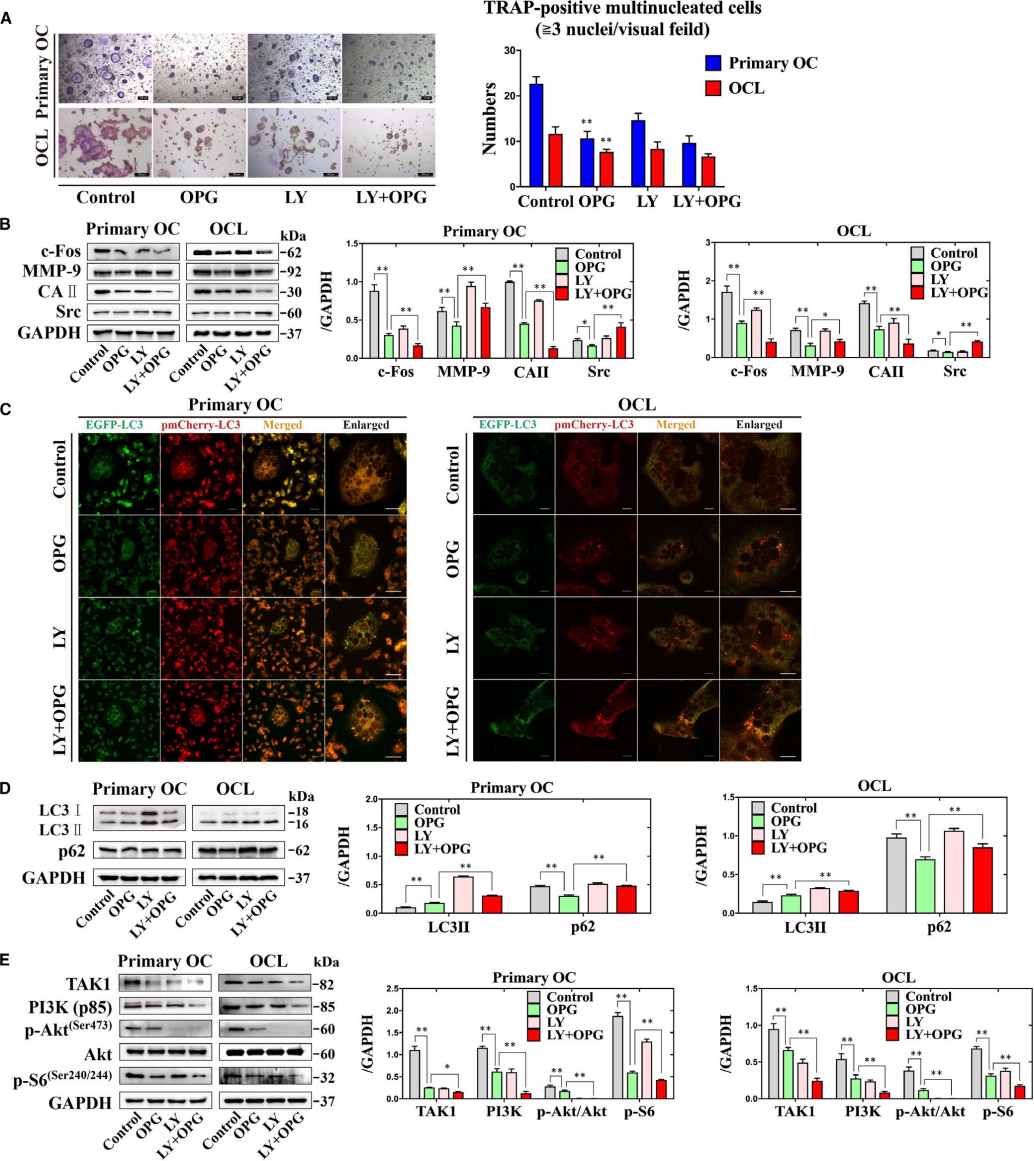
Inhibition of autophagy using the PI3K inhibitor LY294002 can regulate the autophagy in response to OPG‐mediated suppression of osteoclastogenesis in primary osteoclasts and OCLs. A, The formation and number of TRAP‐positive multinucleated cells were counted under different treatments. Magnification × 100. Scale = 100 µm. B, The expression of c‐Fos, MMP‐9, CAⅡ and Src were detected incubation with different treatments. C, EGFP‐pmCherry‐LC3 puncta were observed incubation with different treatments. D and E, The expression of LC3II, p62, the PI3K/Akt and the TAK1/S6 signalling‐related proteins were detected incubation with different treatments. All values are the mean ± SD. ^**^ *P* < .01, ^*^ *P* < .05

LY294002 (LY), the PI3K inhibitor, which can block the formation of phagophore in the early stage of autophagy.^27^, ^28^ There are no different on the number of TRAP‐positive multinucleated cells treatment with LY + OPG group compared with OPG‐only group (Figure 3A). The expression of c‐Fos and CAII were decreased, but the expression of MMP‐9 and Src were increased by treated with LY + OPG group compared with OPG‐only group (Figure 3B). Osteoclasts could be transduced the survival signalling by various stimulus through the PI3K/Akt signalling pathway.^29^ Next, there are observed the accumulation of LC3 puncta by treated with LY294002 + OPG group compared with OPG‐only group (Figure 3C). The expression of LC3II and p62 were increased by treated with LY + OPG group compared with OPG‐only group (Figure 3D). However, the expression of TAK1, PI3K (p85) and p‐S6 (Ser240/244), and the ratio of p‐Akt (Ser473)/Akt were decreased by treated with LY + OPG group compared with OPG‐only group (Figure 3E). These data shown that PI3K inhibitor LY294002 could inhibit autophagy and is associated with OPG‐mediated suppression of osteoclastogenesis.

### 3.4 Continuous activation of c‐Fos could reverse OPG‐mediated suppression of osteoclastogenesis via activation of autophagy

**Figure 4:**
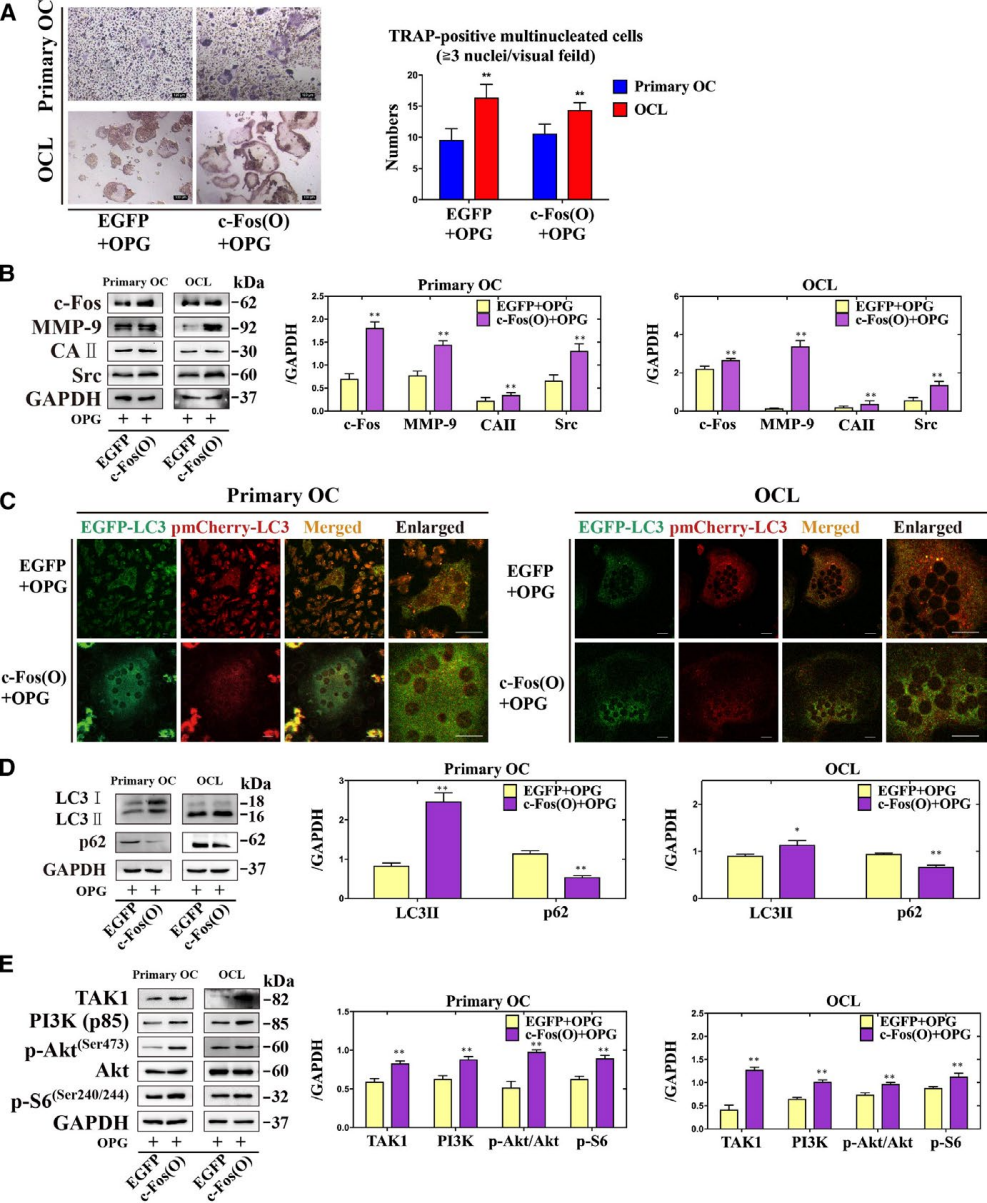
Continuous activation of c‐Fos could reverse OPG‐mediated suppression of osteoclastogenesis via activation of autophagy in primary osteoclasts and OCLs. A, The formation and number of TRAP‐positive multinucleated cells were counted after treatment of OPG with or without overexpression of c‐Fos. Magnification × 100. Scale = 100 µm. B, The expression of c‐Fos, MMP‐9, CAⅡ and Src were detected incubation with different treatments. C, EGFP‐pmCherry‐LC3 puncta were observed incubation with different treatments. D and E, The expression of LC3II, p62, the PI3K/Akt and the TAK1/S6 signalling‐related proteins were detected incubation with different treatments. All values are the mean ± SD. ^**^ *P* < .01, ^*^ *P* < .05

c‐Fos is a key component in osteoclastogenesis.^30^ The number of TRAP‐positive multinucleated cells were increased by treated with overexpression of c‐Fos (c‐Fos[O]) + OPG group compared with EGFP + OPG group (Figure 4A). The expression of c‐Fos, MMP‐9, CAII and Src were increased by treated with c‐Fos(O) + OPG group compared with EGFP + OPG group (Figure 4B). Furthermore, the accumulation of LC3 puncta and the expression of p62 were reduced, but the expression of LC3II, TAK1, PI3K (p85) and p‐S6 (Ser240/244), and the ratio of p‐Akt (Ser473)/Akt were increased by treated with c‐Fos(O) + OPG group compared with EGFP + OPG group (Figure 4C‐E). These data suggested that continuous activation of c‐Fos could rescue the OPG‐mediated suppression of osteoclastogenesis through increasing the autophagy and the PI3K/Akt and the TAK1/p‐S6 signalling pathways.

## 4 DISCUSSION

Osteoclastogenesis requires RANKL to bind to its receptor RANK. OPG compete binding to its decoy receptor RANKL to prevent excessive osteoclastic resorption. Previous study shown that OPG inhibits osteoclastogenesis and bone resorption by enhancing autophagy.^20^ To identify the role of OPG through observing the formation of osteoclastogenesis, the results showed that inhibition of osteoclasts by treated with OPG. RANKL binds to RANK performing the intracellular signalling cascades of osteoclast, including the PI3K/Akt and the TNF receptor associated factors (TRAFs)/TAK1 signalling pathway. TRAFs combine with RANK to initiate osteoclastogenesis‐related signalling, including the PI3K/Akt signalling pathway, thereby activating AP‐1 causing the up‐regulation of c‐Fos.^31^ In addition, activation and maturation of osteoclasts requires transcription factor Src, which is involved in bone resorption and cell adhesion of osteoclast.^32^ Likewise, osteoclasts can secrete hydrolytic enzymes and are associated with extracellular acidification of bone resorption lacuna.^33^ Presented data demonstrated that OPG inhibits osteoclastogenesis through the PI3K/Akt and the TAK1/S6 signalling pathways, while enhancing autophagy.

Osteoprotegerin, as a decoy receptor for TNF‐related apoptosis‐inducing ligand (TRAIL), can attenuate TRAIL‐induced apoptosis by activating the PI3K/Akt signalling pathway.^34^ RANK binds to Src activating the TRAF6 and cannabinoid receptor 1 (Cb1), including the PI3K (p85)/Akt signalling pathway.^35^ RANK combines with TRAF6 activating the IκB kinase (IKK) complex via TAK1, while TAK1 mutant inhibited RANKL‐induced osteoclastogenesis by inactivating of NF‐κB and AP‐1.^36^ The number of osteoclast was reduced treatment with LY294002 by decreasing the expression of NFATc1, not c‐Fos.^37^ In addition, TAK1 plays an essential role in RANKL‐induced osteoclastogenesis. TAK1‐induced cell apoptosis was observed in macrophages, which is dependent on the constitutive autocrine action of TNFα for receptor‐interacting protein 1 (RIP1) activation and reactive oxygen species (ROS) production.^38^ Lacking of TAK1 in monocytes died rapidly, but could be rescued by inhibiting of RIP1 kinase activity with necrostatin‐1 or deletion of *TNFR1*. Interestingly, TNFα‐induced cell death was abrogated by treated with caspases inhibitor or knockdown of RIP3 in TAK1‐deficient mice embryonic fibroblasts.^19^ Thus, LY294002 attenuates osteoclastogenesis by reducing the expression of c‐Fos, Src, MMP‐9 and CAⅡ, blocking autophagy and PI3K/Akt and TAK1/S6 signalling pathways.

**Figure 5:**
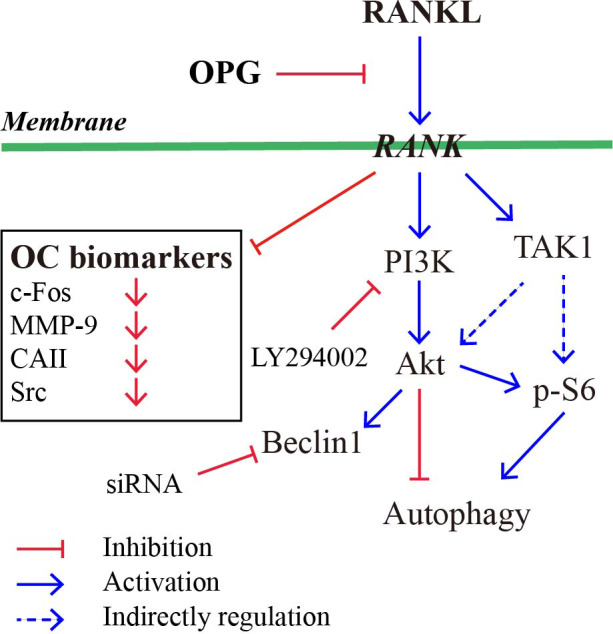
The schematic mode that c‐Fos and Beclin1 are key regulators during OPG‐mediated suppression of osteoclastogenesis. Red arrows indicate inhibitory modification and blue arrows indicate stimulatory modification

Beclin1 have a dual role in RANKL‐induced osteoclastogenesis. Knockdown of Beclin1 attenuates RANKL‐induced osteoclastogenesis by reducing ROS production and NFATc1 expression.^26^ In addition, Beclin1‐mediated autophagy is associated with the Vps34/PI3K (class III) complex in human breast cancer cells.^39^ Activation of autophagy leads to Beclin1 dissociated with TGF‐β‐activated kinase 1‐binding protein 2 (TAB2) to initiate TAK1 during microRNA‐155‐induced osteoclastogenesis.^40^ Furthermore, TAK1 as an upstream activator for the PI3K/Akt signalling pathway in osteoclastogenesis, causing TAK1‐mediated Akt activation, which is essential for the survival of osteoclast survival stimulated by TGF‐β.^41^ Activation of autophagy increased the expression of Beclin1 during OPG‐mediated suppression of osteoclastogenesis. Knockdown of Beclin1 could rescue the activation of c‐Fos and MMP‐9 during OPG‐mediated suppression of osteoclastogenesis, which caused by blocking autophagy and activation the PI3K/Akt signalling pathway.

c‐Fos, is a key regulator of macrophage and osteoclastic lineage, and might provide a critical therapeutic target for bone diseases.^42^ c‐Fos has a dual role in osteoclastogenesis.^43^ Overexpression of c‐Fos could inhibit the maturation of the murine myeloblastic leukaemia cells into macrophages. However, lack of c‐Fos in mice lead to osteopetrosis due impair osteoclast development.^43^ Presented data shown that overexpression of c‐Fos could reverse the formation and function of osteoclasts by increasing the expression of MMP‐9, CAⅡ and Src. In addition, CQ acts as a lysosomal inhibitor and inhibits osteoclastic lysosomal acidification by preventing autophagy.^23^, ^44^ Rap acts as an autophagic activator by inhibiting mTOR, causing attenuation of osteoclast differentiation.^45^, ^46^ The expression of LC3Ⅱ was increased and the expression of p62 was decreased, suggested that overexpression of c‐Fos can reverse autophagy in osteoclastogenesis. Finally, there are presented a schematic model that c‐Fos and Beclin1 are key regulators during OPG‐mediated suppression of osteoclastogenesis, and can dominate the regulatory function in this process (Figure 5).

In summary, the current study has confirmed that c‐Fos has an indispensable role in the regulation of autophagy during OPG‐mediated suppression of osteoclastogenesis. Therefore, c‐Fos appears to provide the new target for exploring the osteoclastogenesis‐related bone diseases.

## CONFLICT OF INTEREST

The authors declare that they have no conflict of interest.

## AUTHOR CONTRIBUTIONS

**Xishuai Tong:** Data curation (equal); Software (equal); Writing‐original draft (equal). **Miaomiao Chen:** Data curation (equal); Software (equal); Writing‐original draft (equal). **Ruilong Song:** Conceptualization (equal); Data curation (equal); Software (equal). **Hongyan Zhao:** Funding acquisition (equal); Project administration (equal). **Jianchun Bian:** Funding acquisition (equal); Project administration (equal); Writing‐review & editing (equal). **Jianhong Gu:** Funding acquisition (equal); Methodology (equal); Project administration (equal); Writing‐original draft (equal); Writing‐review & editing (equal). **Zongping Liu:** Conceptualization (equal); Funding acquisition (equal); Project administration (equal); Writing‐review & editing (equal).

## Data Availability

The data that support the findings of this study are available from the corresponding author upon reasonable request.
